# Structure Formation and Tribological Properties of Mo-Si-B-Hf Electrospark Coatings Based on Mo_2_Ni_3_Si Laves Phase

**DOI:** 10.3390/ma15165613

**Published:** 2022-08-16

**Authors:** Evgenia Igorevna Zamulaeva, Alexander Nikolaevich Sheveyko, Yuri Yurievich Kaplanskii, Evgeny Alexandrovich Levashov

**Affiliations:** Scientific-Educational Center of SHS, National University of Science and Technology “MISiS”, Leninsky Prospect 4, 164, Moscow 119049, Russia

**Keywords:** electrospark deposition, Laves phase, molybdenum silicides, friction and wear, structural transformations, in situ TEM

## Abstract

Coatings were produced on the EP741NP nickel alloy substrates by electrospark deposition (ESD) in argon using an MoSi_2_-MoB-HfB_2_ electrode. In situ high-resolution transmission electron microscopy and X-ray diffraction analysis studies have identified the temperature above which the strengthening Mo_2_Ni_3_Si Laves phase is formed in the coatings. At 25 °C, the coatings with a predominant content of the Laves phase are characterized by enhanced wear resistance, as well as a lower coefficient of friction compared to the non-annealed coatings containing binary silicides. At 700 °C, the EP741NP substrate was characterized by the lowest friction coefficient (Ktr = 0.35), and its wear was approximately at the same level as the wear of both coatings.

## 1. Introduction

The EP741NP alloy is a powder complex alloy used in aircraft and spacecraft engine building [1]. Due to its high strength characteristics and wear and creep resistance, this high-temperature heat-resistant nickel alloy is applied to manufacture gaskets for the detachable connections in liquid propellant rocket engines, as well as disks, shafts and other heavy-duty components operating at temperatures up to 750 °C [2,3].

Durability of the alloy is enhanced using protective coatings. Electrospark deposition (ESD) allows production of high-temperature oxidation-resistant and wear-resistant coatings characterized by high adhesion. Among the broad range of materials potentially suitable for ESD coatings manufacturing, transition metal silicides are especially noteworthy due to their high melting point, high-temperature oxidation resistance, creep resistance, etc. [4]. However, despite MoSi_2_ being resistant to oxidation at temperatures below 1700 °C due to the formation of silica film, it is prone to “pesting” (corrosion involving oxidation at grain boundaries, embrittlement, and self-comminution) in the temperature range of 450–700 °C. An efficient method for inhibiting this phenomenon is to dope the alloy with boron, which results in the formation of compound borosilicate glass, including SiO_2_ and B_2_O_3_ [5,6]. The composite molybdenum disilicide-based ceramics doped with different refractory borides are promising materials for protective coatings. Potanin et al. [7] studied the effect of MoB phase content on the oxidation resistance of MoSi_2_–MoB ceramics at 900 °C. They showed that a composite material with 90 at.% of MoSi_2_ and 10 at.% of MoB was characterized by the strongest oxidation resistance. After testing the ceramics with these compositions as electrode materials for electrospark deposition of EP718ID nickel alloy in air [8], the same ceramics containing 10% MoB were found to have the best properties. An analysis of the coating demonstrated that its structure consists of a nickel-based solid solution with hP12-type Mo_2_Ni_3_Si ternary silicide distributed over it. Later, the electrode material was additionally doped with HfB_2_ [9,10]. Similar to molybdenum diboride, hafnium diboride prevents the intense oxidation of MoSi_2_. A specific feature of electrospark deposition is that, during the process, both the electrode material being deposited and the substrate are involved in phase formation of the protective layer. In [11], coatings were deposited onto EP741NP nickel alloy in argon using MoSi_2_–MoB and MoSi_2_–MoB–HfB_2_ electrodes. The coatings consisted of MoSi_2_ and Mo_5_Si_3_ binary silicides and a γ-Ni-based solid solution. Oxidation resistance tests at 900 °C revealed that the Mo_2_Ni_3_Si Laves phase is formed in the coatings after 5 h, and its content increases with annealing duration.

The Laves phases of transition metals with the AB_2_-type crystal structure are of special interest due to their high hardness, strength, corrosion resistance, and long-term stability at temperatures above 1000 °C [12,13,14].

In 2003–2019, a large amount of research was carried out to study composite materials and coatings containing ternary metal silicide Mo2Ni3Si as the main phase [15,16,17,18,19,20,21,22,23,24,25,26,27]. The interest in these composites is due to the fact that, compared to binary metal silicides, the Mo_2_Ni_3_Si phase has a higher toughness while maintaining high hardness [15,16,17]. The tribological behavior of Mo_2_Ni_3_Si-based composites was studied under dry friction conditions [15,16,17,18,20,21,22,23,24,25,26] and in corrosive environments [19]. The samples were tested at room temperature [16,18,19,20,21,22,23,24,25,26] and at elevated temperatures [15,17]. In all cases, the tests showed high wear resistance which is explained by the simultaneous high hardness of Mo_2_Ni_3_Si dendrites and the toughening effect of the ductile interdendritic media [21,22]. The dominant wear mechanism of these composites is considered to be alternating processes of preferential abrasion of the interdendritic media and spalling of the Mo_2_Ni_3_Si primary dendrites [21,22].

To obtain samples based on the Mo_2_Ni_3_Si phase, the mentioned research used methods such as laser cladding [15,16,17,18,19,20,26,27], arc-melting process [23,24,25], and laser-melting process [21,22], which, similar to the elektrospark deposition method, are associated with the material melting. However, an important difference between ESD and these methods is the short-term effect of electric discharges and the small extent of the melt region. This causes significantly higher cooling rates and strongly nonequilibrium crystallization conditions. Therefore, phase formation does not have time to complete during ESD, and annealing is required for the formation of the Mo_2_Ni_3_Si phase, while in the mentioned studies, the Mo_2_Ni_3_Si phase is directly formed as large dendrites at crystallization.

In this study, we determined the temperature of Laves phase nucleation in coatings applied by ESD onto EP741NP nickel alloy in argon using a MoSi_2_–MoB–HfB_2_ electrode and compared the tribological properties of the coatings containing dispersed Laves phase precipitates with coatings based on binary silicides.

## 2. Materials and Methods

Disc-shaped samples (ø20 × 5 mm) produced from EP741NP alloy by selective laser sintering on an EOS400 device were used as a substrate (cathode). The composition of the alloy complies with the State Standard GOST 52802-2007 (Ni matrix; C, 0.02–0.06 wt.%; Cr, 8.0–10.0 wt.%; Ti, 1.6–2.0 wt.%; Al, 4.8–5.3 wt.%; Mo, 3.5–4.2 wt.%; Nb, 2.4–2.8 wt.%; Co, 15.0–16.5 wt.%; W, 5.2–5.9 wt.%; Mg, 0.02 wt.%; B, 0.015 wt.%; Ce, 0.01 wt.%; Hf, 0.1–0.4 wt.%; and Zr, 0.015 wt.%).

A 75% MoSi_2_—5% MoB—20% HfB_2_ electrode (anode) was fabricated by self-propagating high-temperature synthesis (SHS) using the technique of forced SHS pressing [9] and was shaped as a rectangular rod (40 × 4 × 4 mm).

Pulsed electrospark deposition was carried out in a 0.25 L chamber in argon (flow rate, 1 L/min) on an Alier-Metal 303 setup (pulse energy E, 0.048 J; current, 120 A; single pulse duration, 20 µs; and pulse repetition frequency, 3200 Hz). Voltage drop across the electrospark interval was 20 V. The vibration frequency of the anode electrode was 600 Hz.

A number of coated samples were annealed in a VE-3-16 shaft-type vacuum electric furnace at 900 °C and 0.013 Pa for 3 h.

The microstructure of the samples was studied on a Hitachi S-3400N scanning electron microscope equipped with a NORAN energy-dispersive X-ray spectroscopy (EDS) module.

The mechanisms of nucleation and growth of dispersed precipitates of the Laves phase were studied in situ by high-resolution transmission electron microscopy (HRTEM) and electron beam diffraction on a JEM-2100 microscope (Jeol, Tokyo, Japan) using a Gatan 652 in situ heating holder (Gatan, Inc., Pleasanton, CA, USA). Ultrathin coating foils were manufactured on a PHIPS II two-beam ion etching system (Gatan, Inc., Pleasanton, CA, USA). Changes in the structure were recorded every 20–25 min during the isothermal exposure at temperatures of 400, 500, 600, 700, 800, and 900 °C. Between the exposures, the foils were heated at a rate of 100 °C/min.

The phase composition of the samples was studied by X-ray diffraction (XRD) analysis using monochromatic Cu-Kα radiation on a D8 DISCOVER diffractometer (stepwise scanning in the range 2θ = 10 ÷ 110°, scan step, 0.1°; exposure duration per point, 6 s). The resulting spectra were analyzed using the JCPDS database.

In order to determine the temperature of formation of the primary Laves phase crystals, the coatings were annealed at 600, 700, and 800 °C in a SNOL 1.1,6/12-M3 electric furnace prior to XRD analysis. The samples were exposed to the target temperature for 3 h.

The measurements of hardness, H, and elasticmodulus, E, were performed by the load-depth-sensing nanoindentation method using a nano hardness tester (CSM Instruments) equipped with a Berkovich diamond indenter tip calibrated against fused silica (ASTM E2546-17) at a load of 10 mN.

The tribological properties were measured in compliance with the international standards ASTM G 99-959 and DIN 50324 on a high-temperature tribometer (CSM Instruments, Switzerland) using a pin-on-disk scheme. An Al_2_O_3_ ball, 6 mm in diameter, was used as a counterbody. The linear sliding speed of the counterbody was chosen to be 10 cm/s; load was 5 N. At constant heating (25 °C and 700 °C) the sliding distance was 300 m. In the case of dynamic heating from 25 °C to 700 °C, the average rate of temperature elevation was 20 °C/min; the sliding distance was 190 m. Temperature was controlled using a thermocouple whose temperature sensing junction was attached to the center of a steel plate where the analyzed sample was mounted. Both the coefficient of friction (Kfr) and temperature were continuously recorded using the InstrumX software at the sliding wear tests. The wear rate of the samples was calculated using the Equation (1):

W = (s × L)/(H × l)(1)

where W is the wear rate, mm^3^∙N^−1^·m^−1^;

L is the circumference, mm;

s is the cross-sectional area of the wear groove, mm^2^;

H is the load, N; and

l is the sliding distance, m.

The wear scar area of the ball was measured using the graduated scale of the optical microscope. The wear track profile and roughness of the samples were measured on a Veeco WYKO NT 1100 optical profiler.

## 3. Results and Discussion

**Figure 1:**
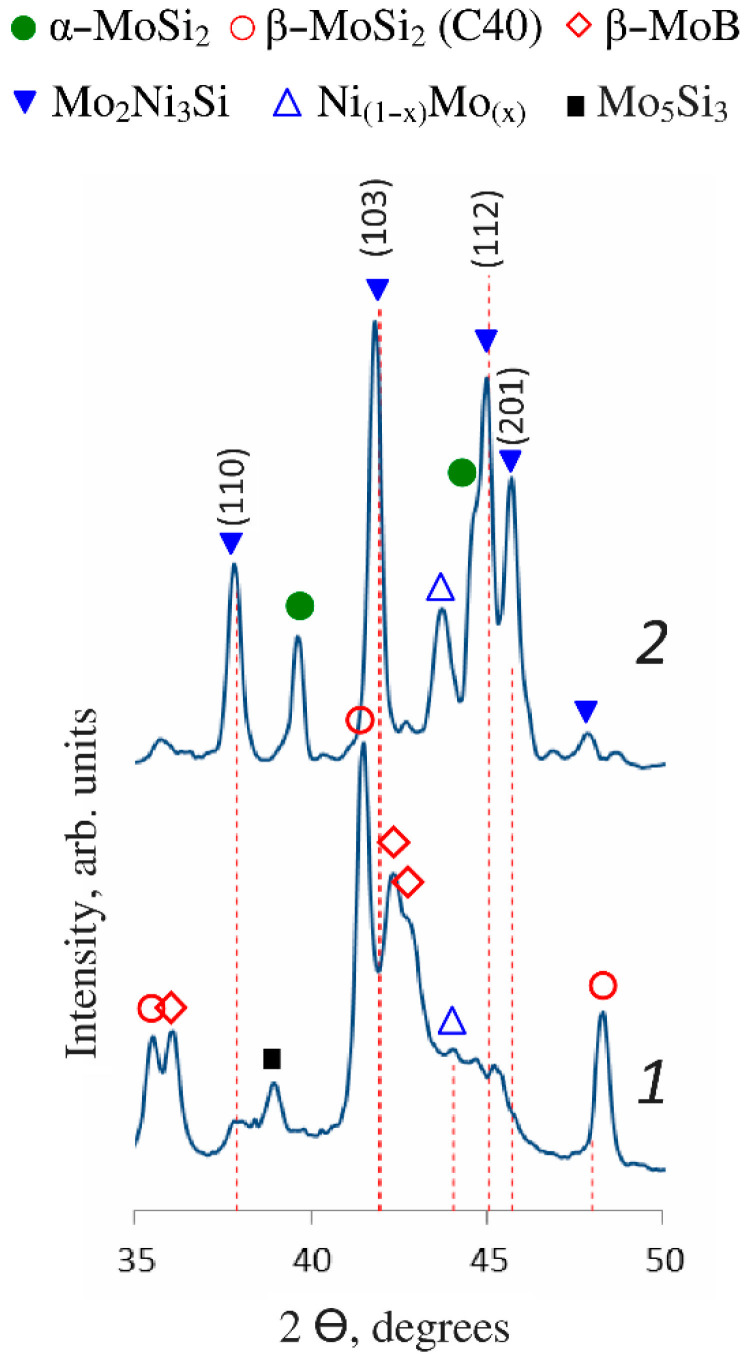
Regions of the XRD patterns of the coating samples deposited using the MoSi_2_-MoB-HfB_2_ electrode in the as-deposited state (1) and after vacuum annealing at 900 °C (2). Dashed lines correspond to the Mo_2_Ni_3_Si phase (ICDD database, card No. 01-089-5030).

Figure 1 shows the regions of XRD patterns for the coating samples immediately after the electrospark deposition (the as-deposited state) and after additional vacuum annealing. For the annealed coatings, we identified strong diffraction peaks corresponding to the Mo_2_Ni_3_Si Laves phase with the MgZn_2_-type crystal structure. Simultaneously with the increasing intensity of reflections from the γ-Ni-based solid solution phase, the disappearance of the peaks belonging to the Mo_5_Si_3_ and β-MoSi_2_ phases indicates that molybdenum silicides were spent to form the thermodynamically stable Mo_2_Ni_3_Si Laves phase [13] which became the main phase in the coating. The metastable high-temperature phase gives way to the low-temperature α-MoSi_2_ phase.

The XRD data have shown (Table 1) that the total content of MoSi_2_ and Mo_5_Si_3_ silicides in the as-deposited coating is 62%.; β-MoB phase, γ-Ni-based solid solution, and hafnium boride HfB_2_ are also present. After annealing, the Mo_2_Ni_3_Si Laves phase becomes the main phase (68%), and the γ-Ni content increases from 6 to 11%. The α-MoSi_2_ phase appears instead of the metastable β-MoSi_2_ phase, but its content is twice as low since some of molybdenum disilicide is spent to form the Mo_2_Ni_3_Si Laves phase.

Due to the typical processing of the ESD method high crystallization rate, it can be assumed that any resulting phase may contain dissolved components in excess of their solubility. Although the electrode material contains 20% HfB_2_, only traces of HfB_2_ are detected in the coating. We can assume that the HfB_2_ is predominantly dissolved in the MoSi_2_ phase. According to the XRD data, the MoSi_2_ phase is the main phase in the as-deposited coating. The subsequent vacuum annealing accelerates the processes of diffusion and dissolution in the coating; therefore, hafnium and boron possibly dissolve in the Laves phase.

**Figure 2:**
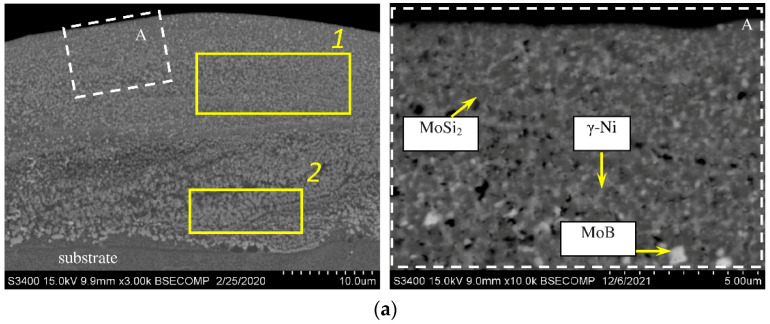

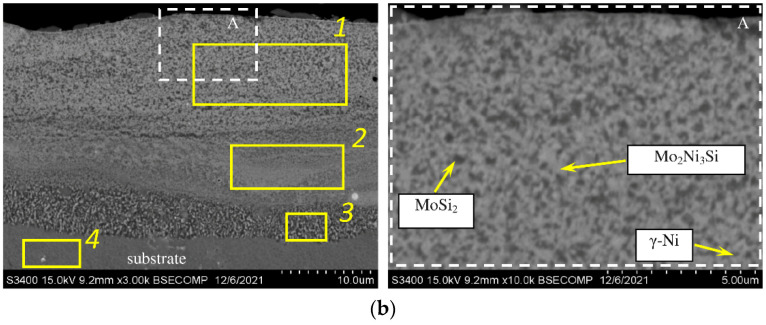
Microstructure of the coating deposited using the MoSi2–MoB–HfB2 electrode: in the as-deposited state (**a**) and after vacuum annealing (**b**).

Figure 2 shows the SEM images of the microstructure of cross-sections of the coated samples before and after vacuum annealing. Both coatings are ~23–26 µm thick and have no signs of pores or cracks. In the electrode material, MoSi2 (the dark phase) and MoB (the light phase) are accurately determined by EDS [7,9,11]. At the ESD, the short duration of the electrical pulse allows an extremely rapid solidification of the deposited electrode material and results in an exceptionally fine-grained, homogeneous coating that approaches (and with some materials, actually is) an amorphous structure [28]. That is why, based on the XRD, SEM, and EDS data, for both the electrode and the coating, the dark gray grains (Figure 2a, inset A) correspond to the MoSi_2_ and Mo_5_Si_3_ phases; light-colored inclusions are the MoB phase, and gray inclusions correspond to the γ-phase of nickel-based solid solution. After the annealing, the coating composition and structure are altered due to diffusion of nickel and other elements contained in the substrate towards the surface (Figure 2b): a transition zone is formed between the coating and the substrate. The light-colored particles (Figure 2b, inset A) can be identified as the Mo_2_Ni_3_Si Laves phase, which is distributed in the gray matrix of the nickel-based solid solution. The dark gray grains correspond to the MoSi_2_ molybdenum disilicide phase.

Table 2 summarizes the EDS data for different regions shown in Figure 2. In the coating, the contents of elements that were originally present in the substrate (Al, Ti, Cr, Co, and Ni) increase after annealing compared to the as-deposited coating (region 1). Nickel concentration rises from 6.5 at.% to 29 at.%.

While the composition and structure of the near-boundary zone of the as-deposited coating (Figure 2a, region 2) slightly differs from the main coating area, two regions can be clearly distinguished between the coating and the substrate after annealing (Figure 2b): the region with a higher chromium content (region 2) and the one with a higher aluminum content (region 3).

**Figure 3:**
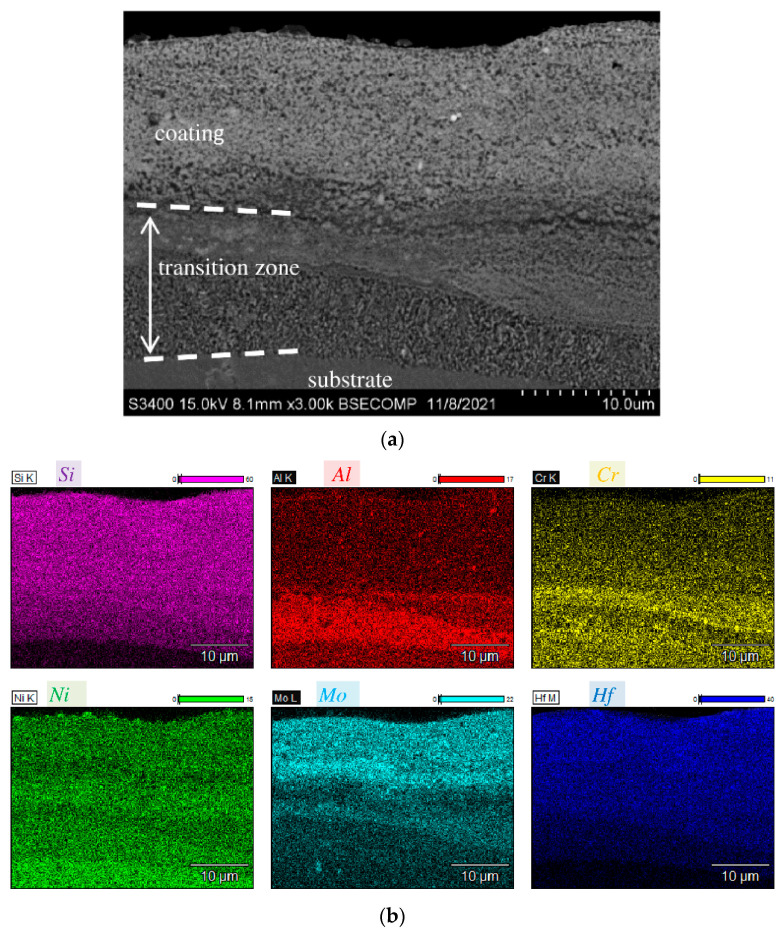
Cross-sectional SEM image (**a**) and corresponding EDS elemental maps (**b**) of the coating after vacuum annealing.

Maps of element distribution in the annealed coating (Figure 3) confirm that a wide transition zone is formed due to diffusion of elements contained in the substrate deep into the coating. Concentration of aluminum at the interface increases, followed by a rise in chromium concentration.

**Figure 4:**
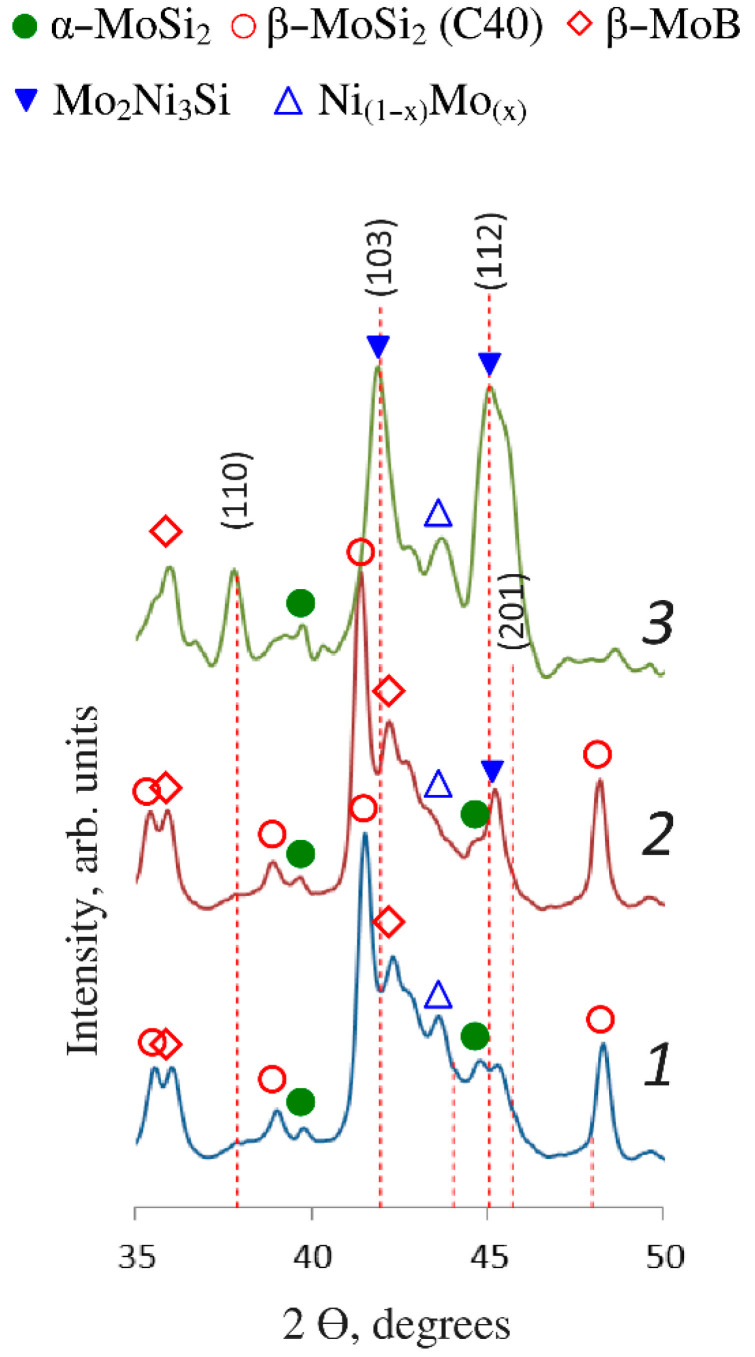
Regions of the XRD patterns of the coating after annealing at 600 °C (1), 700 °C (2), and 800 °C (3). Dashed lines correspond to the Mo_2_Ni3Si phase (ICDD database, card No. 01-089-5030).

In order to identify the conditions under which the Mo_2_Ni_3_Si phase is formed, the as-deposited coating produced using the MoSi_2_–MoB–HfB_2_ electrode, was heated to different temperatures. Figure 4 shows the regions of the XRD spectra of the samples. As shown in Table 3, the phase composition changed after annealing in air at 600 °C: the silicide phase Mo_5_Si_3_ disappears. The content of the β-MoSi_2_ phase is 53%; the β-MoB phase, 25%; the Ni_(1−x)_Mo_(x)_ phase, 10%; and the α-MoSi_2_ phase, 10%. The Laves phase is formed at 700 °C and its concentration is 10% (band 2). At 800 °C, the content of the Laves phase increases to 35%, while there are no picks corresponding to the β-MoSi_2_ phase (band 3). At 600 °C and 700 °C, the coatings contain traces of hafnium oxide. At 800 °C, the amount of oxide phases in the coating increases.

**Figure 5:**
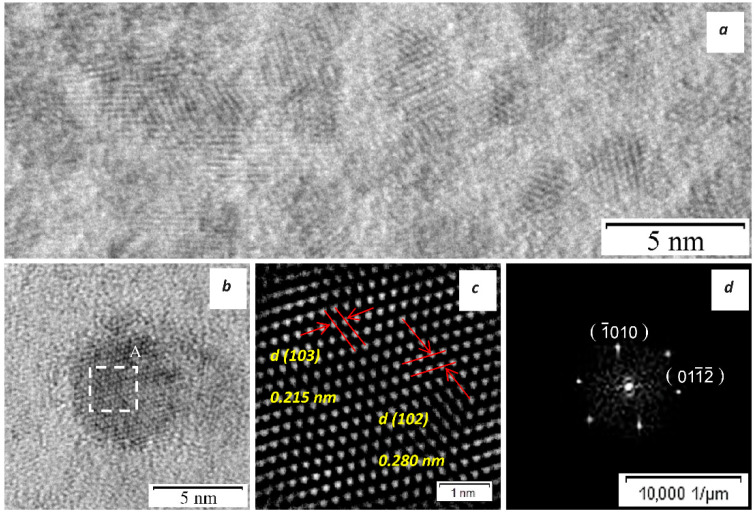
(**a**) The HRTEM image of the coating structure heated up to 800 °C; (**b**) the [$\bar{2}4\bar{2}$3] HRTEM image of the Mo_2_Ni_3_Si phase pre-precipitate with corresponding IFFT (**c**) and FFT (**d**) patterns from region A.

An in situ HRTEM study of the structural phase transformations in the coating occurring upon heating to 900 °C revealed that nucleation and growth of pre-precipitates of the Mo_2_Ni_3_Si Laves phase begin at 700 °C and actively occur at 800 °C (Figure 5a). The nucleation of the Laves phase presumably results from the decomposition of a supersaturated nickel solid solution in the MoSi_2_ phase.

Figure 5b shows a characteristic image of the atomic structure of a 5-nm pre-precipitate. The inverse fast-Fourier transform (IFFT) and fast-Fourier transform (FFT) of the atomic structure of a pre-precipitate from the analyzed region A (Figure 5c,d) oriented along the zone axis [$\bar{2}4\bar{2}$3] confirms that Mo_2_Ni_3_Si Laves phase nanoparticles are formed in the MoSi_2_-based coating (lattice parameters: a = 0.4681 nm, c = 0.7523 nm). The experimental values of lattice parameters of the excessive phase differ somewhat from the tabular data (a = 0.4745 nm, c = 0.7578 nm) because of the nonstoichiometric composition. Further heating to 900 °C led to an increase in the volume percentage of Laves phase pre-precipitates.

The process of cooling down the ultrathin coating foil from 900 °C to room temperature is accompanied by rapid coalescence of pre-precipitates and formation of Mo_2_Ni_3_Si nanoparticles sized up to 100 nm, which was described in [11].

**Figure 6:**
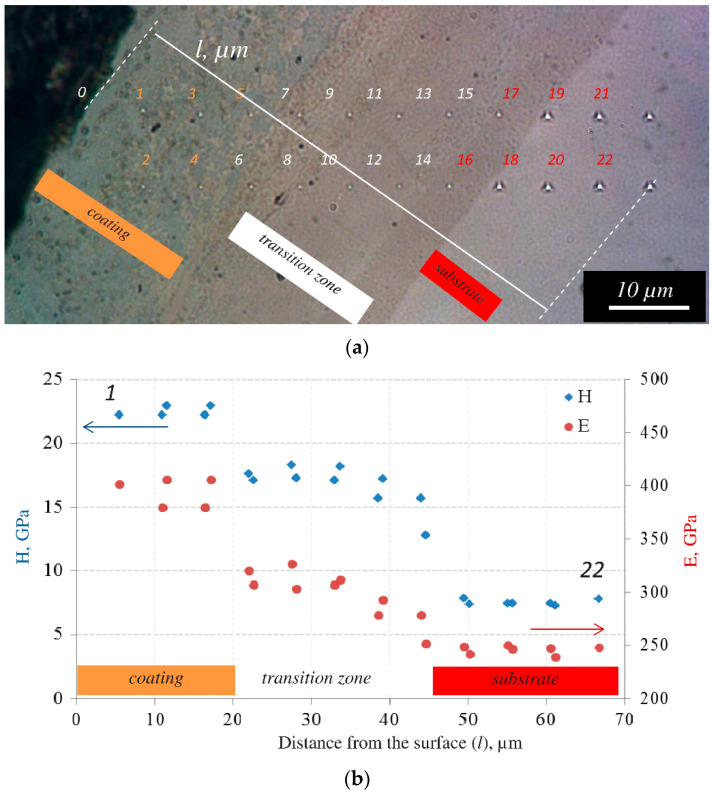
Cross-sectional optical image of the coating based on the Mo_2_Ni_3_Si Laves phase, which shows the positions of indents (**a**) and the distribution of H and E values over coating thickness (**b**).

Figure 6 shows the distribution of mechanical properties over the thickness of an annealed coating based on the Mo_2_Ni_3_Si Laves phase. Two series of the measurements were performed in direction from the coatings surface to the nickel alloy substrate at a distance of 10 μm from each other while moving the sample with a step of 8 μm (Figure 6a). Hardness (H) and the Young’s modulus (E) of the vacuum-annealed coating were 22.4 GPa and 394 GPa, respectively. These values were 7% and 20%, respectively, higher than those of the as-deposited coating (H = 20.9 GPa and E = 317 GPa) [11]. In the transition zone, the H and E values decreased to 17.1 and 310 GPa, respectively. The hardness of the MoSi_2_ (1320–1550 HV) and Mo_5_Si_3_ (1200–1320 HV) phases [29] are higher than the hardness of the Laves phase (1100 HV) [21]. We suppose that this effect is observed due to an increase in the coating density (decreasing their porosity). This occurs as a result of nickel diffusion and grain growth at annealing.

The following samples were tested to identify the effect of phase composition on tribological properties of the coatings: (1) as-deposited coatings (immediately after ESD); (2) coatings after vacuum annealing (based on the Mo_2_Ni_3_Si Laves phase); and (3) the uncoated EP741NP substrate. The phase composition of the coatings is listed in Table 1.

**Figure 7:**
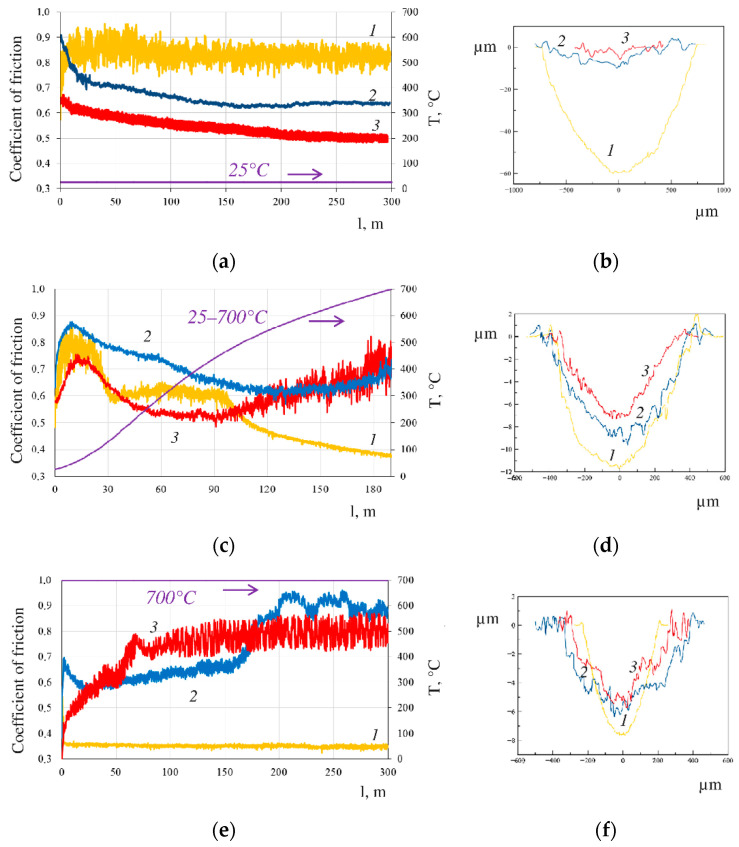
Friction coefficient of the samples at different temperatures as a function of distance (**a**,**c**,**e**) and the respective wear track profiles (**b**,**d**,**f**): 1—uncoated substrate; 2—as-deposited coating; and 3—annealed coating.

Figure 7 shows the dependences of the coefficient of friction at different temperatures (a, c, e) and the corresponding wear track profiles (b, d, f). At 25 °C, the coefficient of friction of the uncoated nickel substrate decreases from 0.87 to 0.83 at sliding distances > 100 m and then remains unchanged until the tests are completed. After Kfr of the as-deposited and annealed coatings reaches the maximum values (0.9 and 0.65, respectively), it decreases to 0.63 and 0.5, respectively. In the dynamic mode, when temperature is increased from 25 °C to 700 °C (Figure 7c,d), all the bands pass through the maximum at the initial stage of the tests (at 60–80 °C). Then, at sliding distances >35 m, Kfr of nickel alloy decreases from 0.78 to 0.59 (at 150 °C) and is relatively stable until the sliding distance of 95 m (at 470 °C); the average Kfr value is ~0.6. At sliding distances >95 m, the plateau ends and Kfr gradually declines to reach 0.37. Changes in Kfr of the as-deposited coating until the sliding distance of 120 m (in the temperature range of 25–50 °C) are stronger than those for the uncoated substrate and the annealed coating strengthened by the Laves phase. Meanwhile, Kfr gradually descends to its minimal value (0.62) at a sliding distance of 130 m (T = 570 °C). Kfr then gradually increases to 0.7. Kfr of the annealed coating at a sliding distance of 90 m and T = 200 °C has a minimal value of 0.51 and then begins to increase. Starting with a sliding distance of 140 m (T = 590 °C) and until the end of the experiment, the Kfr values roughly coincide with the findings for the as-deposited coating. Being moderate during the first half of the sliding distance, the oscillation amplitude of Kfr of the annealed coating begins to increase after the sliding distance of 90 m and reaches 0.8, which is several-fold higher than the oscillation amplitude of Kfr for the as-deposited coating. At 700 °C, the Kfr value of nickel alloy is 0.35 at all the sliding distances, the oscillation amplitude is approximately 0.005. The Kfr value of the as-deposited coating initially decreases from 0.68 to 0.56 at a sliding distance of 25 m, and then gradually increases to 0.66. During the second half of the sliding distance, Kfr rapidly increases. At sliding distances of 220 and 250 m, Kfr has local maxima (~0.95); its final value is 0.88. The shape of the dependence curves is different at 700 °C: Kfr of the coating strengthened by the Laves phase reaches 0.78 during the first 140 m of sliding distance and remains at this level until the end of the experiment. The steady-state mode is attained with an oscillation frequency of Kfr of ~0.07.

**Figure 8:**
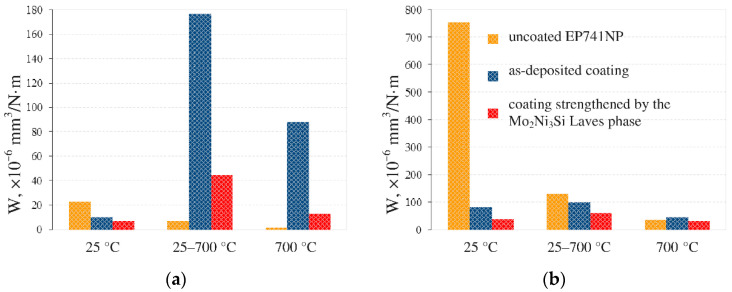
Wear rate of a friction pair at different temperatures: (**a**) for Al_2_O_3_ balls and (**b**) for the substrate (uncoated and with coatings deposited onto it).

The wear rate values were calculated for each tribological pair after testing in each analyzed temperature mode (Figure 8). At all experimental conditions, the wear-resistant of coatings depends less significantly on the temperature regime than the wear-resistant of the nickel alloy. The difference in the wear resistance of the coatings based on binary silicides and the Laves phase is 2–1.5 times due to their different phase homogeneity. The decrease in the wear of the nickel alloy with increasing temperature can be explained by the appearance of oxides of the Ni alloy components, which probably play the role of a solid lubricant, which allows the tribological pair to slide almost without wear. At 25 °C, the wear rate of dispersion-strengthened coating with a high content of the Mo_2_Ni_3_Si Laves phase was two-times lower than that of the as-deposited coating and 20-fold lower than that of the uncoated substrate. At 700 °C, the wear rate values were close for nickel alloy and the coatings.

**Figure 9:**
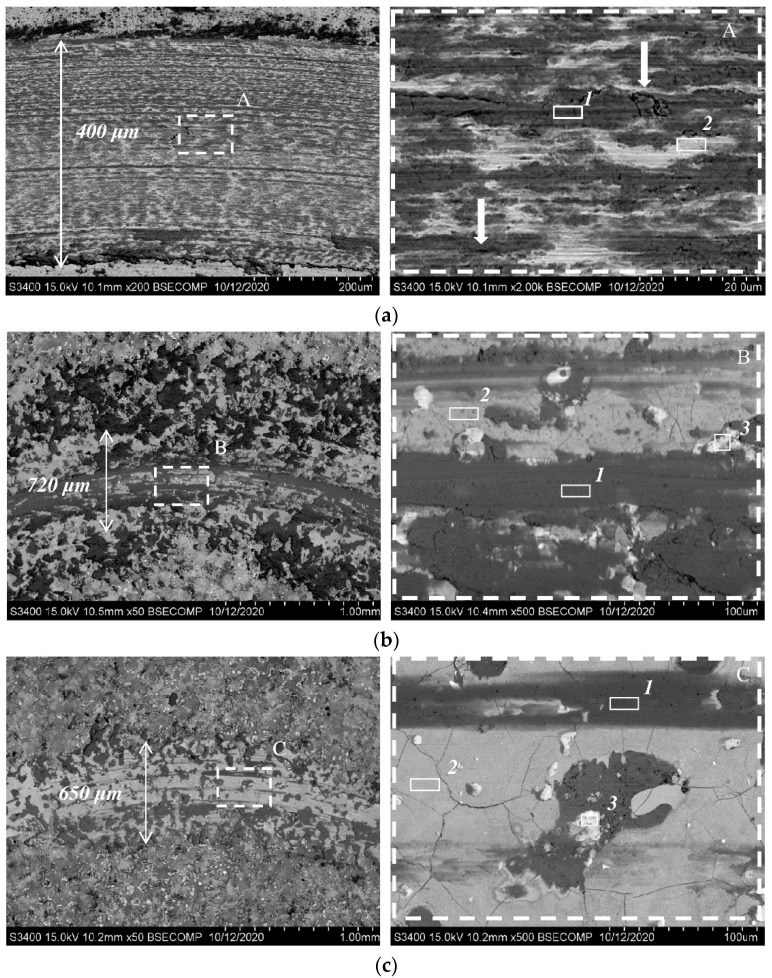
SEM images of the wear tracks after tribological tests at 700 °C for (**a**) uncoated substrate; (**b**) as-deposited; and (**c**) vacuum-annealed coating.

Figure 9 shows the SEM images of wear tracks formed during the tests at 700 °C; Table 4 summarizes the EDS data for different areas of the wear tracks (Figure 9, inset A, B, C). The darker regions in the SEM images correspond to wear products that mostly consist of oxides (region 1) and fill the surface imperfections. For the uncoated sample and the as-deposited coating, the wear products are also present and look like piles next to the wear track. The high content of aluminum among the wear products in the as-deposited coating results from the fact that the counterbody preferentially undergoes abrasion. No piles next to the wear track were observed for the dispersion-strengthened coating; the content of wear products in the wear track was found to be lower. The light-colored areas in the SEM images correspond to the material of the as-deposited alloy or the coatings. The light-colored areas were found to contain a noticeable amount of oxygen due to the formation of an oxide film.

**Figure 10:**
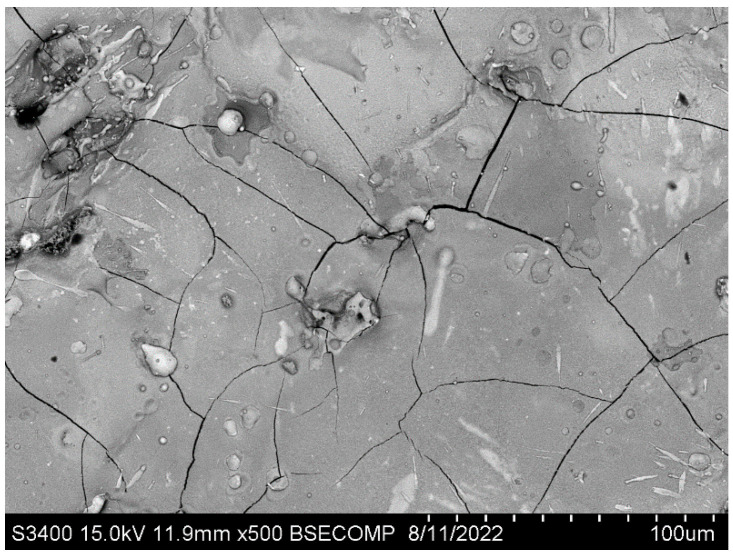
Top-view SEM image of the coating in the as-deposited state.

The wear track of the uncoated nickel alloy contains many parallel grooves, indicating that abrasive wear via the microcutting mechanism predominates (Figure 9a). Light-colored sections of the coatings contained neither these regions nor deformation marks. Surface microcracks are visible (Figure 9b,c), which is typical of hard electrospark coatings; however, microcracks of this type usually have no significant effect on their tribological properties [30,31]. Figure 10 demonstrates the coating’s surface covered by a network of cracks in the as-deposited state.

Hence, the dispersion-strengthened coating with the Laves phase as the predominant component had the lowest coefficient of friction (Kfr = 0.5) at T = 25 °C, while at 700 °C the lowest coefficient of friction (Kfr = 0.35) was observed for the substrate made of uncoated nickel alloy. At T = 25 °C, the wear rate of the dispersion-strengthened coating was twice as low as that of the as-deposited coating and 20-fold lower than that of the uncoated substrate.

## 4. Conclusions

1. High-resolution transmission electron microscopy and X-ray diffraction phase analysis have shown that the formation of the Mo2Ni3Si Laves phase in the initial coatings begins at 700 °C and actively proceeds at 800 °C. The content of the Laves phase increases with temperature and annealing duration.
2. Pin-on-disc tribological tests have been conducted at T = 25 °C, under dynamic heating from 25 to 700 °C, and at T = 700 °C. At 25 °C, the coating with a predominant content of the Laves phase was characterized by a reduced friction coefficient (Ktr = 0.5) and enhanced wear resistance with specific wear two-times lower than that of the initial coating and 20-times lower than that of the EP741NP alloy. At 700 °C, the lowest value of the friction coefficient (Ktr = 0.35) was noted for the EP741NP substrate, and its wear was approximately on the same level as the wear of both coatings.

## Figures and Tables

**Table 1:** Phase composition of the coating.

Phase	Structural Type	State			
		As-Deposited		After Annealing	
		wt.%	Lattice Parameters, nm	wt.%	Lattice Parameters, nm
β-MoSi2 (C40-type)	hP9/3	43	a = 0.4618 c = 0.6521		
α-MoSi2 (C11b-type)	tI6/2			19	a = 0.3203 c = 0.7846
Mo2Ni3Si (C14-type)	hP12/1			68	a = 0.4734 c = 0.7581
Mo5Si3 (D8.8-type)	hP16/2	19	a = 0.7257 c = 0.4917	–	
β-MoB (Bf-type)	oC8/2	27	a = 0.3152 b = 0.8474 c = 0.3073	2	–
γ- Ni (A1-type)	cF4/1	6	a = 0.3578	11	a = 0.3578
HfB2 (C32-type)	hP3/4	5	a = 0.3092 c = 0.3367		

**Table 2:** EDS data for the coatings produced using the MoSi_2_–MoB–HfB_2_ electrode before and after annealing (Figure 2).

Analyzed Region	Element, at.%										
	B	Al	Si	Ti	Cr	Co	Ni	Mo	Hf	Nb	W
As-deposited state											
1	15.5	0.0	51.0	0.0	0.9	0.0	5.6	23.6	3.4	0.0	0.0
2	20.2	0.0	44.7	0.0	1.4	2.8	8.4	19.7	2.8	0.0	0.0
After annealing											
1	13.3	1.2	25.1	1.2	3.7	6.5	29.0	15.0	5.0	0.0	0.0
2	4.7	8	21.9	2.3	10.1	12.7	32.1	6.3	1.9	0.0	0.0
3	3.9	15.7	17.5	3	7.1	11.9	35.5	2.9	0.3	2.2	0.0
4	0.0	10.2	0.0	2.4	10	16.2	55.5	2.5	0.0	1.3	1.9

**Table 3:** Phase composition of the coating after annealing at indicated temperatures.

Phase	Structural Type	Temperature					
		600 °C		700 °C		800 °C	
		wt.%	Lattice Parameters, nm	wt.%	Lattice Parameters, nm	wt.%	Lattice Parameters, nm
β-MoSi2 (C40-type)	hP9/3	53	a = 0.4617 c = 0.6507	51	a = 0.4613 c = 0.6520	-	-
α-MoSi2 (C11b-type)	tI6/2	10	-	2	-	2	-
Mo2Ni3Si (C14-type)	hP12/1	-	-	10	-	35	a = 0.4738 c = 0.7569
β-MoB (Bf-type)	oC8/2	25	a = 0.3160 b = 0.8467 c = 0.3081	26	a = 0.3159 b = 0.8465 c = 0.3080	21	a = 0.3150 b = 0.8437 c = 0.3079
γ- Ni (A1-type)	cF4/1	10	a = 0.3584	7	a = 0.3585	8	a = 0.3579
HfO2 (C43-type)	mP12/3	2	-	4	-	9	-
MoNiO4						25	a = 1.0197 b = 0.9183 c = 0.7044 β = 10.6668

**Table 4:** EDS data for different areas of the wear tracks (Figure 9).

Inset	Analyzed Region	Element, at.%									
		O	B	Al	Si	Ti	Cr	Co	Ni	Mo	Hf
A	1	54.3	0.0	5.3	0.0	1.5	5.7	5.9	27.3	0.0	0.0
	2	15.8	0.0	6.7	0.0	1.8	8.7	14.4	52.6	0.0	0.0
B	1	63.9	0.0	22.3	6.5	0.2	0.5	0.0	2.2	2.7	1.7
	2	27.2	0.0	2.6	21.5	0.8	3.5	4.8	22.1	13.3	4.2
	3	54.9	13.5	9.7	0.0	0.0	0.0	0.0	1.8	0.0	20.1
C	1	66.7	0.0	6.8	9.7	0.3	0.6	1.4	7.9	4.1	2.5
	2	11.9	0.0	1.6	19.8	0.8	2.3	3.8	35.5	17.6	6.7
	3	57.3	15.4	6.8	0.0	0.0	0.0	0.0	0.0	0.0	20.5

## Data Availability

Not applicable.
